# Anaprazole-based versus rabeprazole-based quadruple therapy for *Helicobacter pylori* eradication: a prospective, multicenter, randomized controlled trial

**DOI:** 10.3389/fcimb.2026.1972507

**Published:** 2026-10-14

**Authors:** Xiujuan Wang, Haocheng Wang, Yan Wang, Lu Shen, Guiqin Li, Jijun Zhu, Changhui Li, Jiahuan Gao, Zhenyu Zhang

**Affiliations:** 1 Department of Gastroenterology, The Anqing 116 Hospital, China RongTong Medical Healthcare Group Co. Ltd., Anqing, China; 2 Department of Gastroenterology, Nanjing First Hospital, Nanjing Medical University, Nanjing, China; 3 Department of Gastroenterology, The First People’s Hospital of Suqian, Suqian, China; 4 Department of Gastroenterology, Changzhou Traditional Chinese Medicine Hospital, Changzhou, China; 5 Department of Gastroenterology, Lianshui County People’s Hospital, Huai’an, China

**Keywords:** amoxicillin, anaprazole, bismuth-containing quadruple therapy, furazolidone, *Helicobacter pylori*

## Abstract

**Background:**

To compare the efficacy and safety of a bismuth-containing quadruple therapy comprising the novel proton pump inhibitor (PPI) anaprazole versus the classical PPI rabeprazole, both combined with amoxicillin and furazolidone, for *Helicobacter pylori* (*H. pylori*) eradication.

**Methods:**

A prospective, multicenter, randomized, open-label, non-inferiority design was employed. Consecutive treatment-naïve patients positive for *H. pylori* who visited the gastroenterology departments of four hospitals in Jiangsu Province between August 2025 and February 2026 were enrolled. Eligible patients were randomly assigned in a 1:1 ratio to two groups: the anaprazole group (anaprazole 20 mg, amoxicillin 1000 mg, furazolidone 100 mg, and colloidal bismuth pectin 300 mg, twice daily) and the rabeprazole group (rabeprazole 20 mg, amoxicillin 1000 mg, furazolidone 100 mg, and colloidal bismuth pectin 300 mg, twice daily). Both groups received a 14-day treatment course. *H. pylori* eradication was assessed by ¹³C-urea breath test at 4 weeks after the completion of therapy.

**Results:**

A total of 320 patients were enrolled, with 160 in each group. In the intention-to-treat (ITT) analysis, the eradication rate was 81.9% (95% CI: 75.2% to 87.1%) in the anaprazole group and 80.0% (95% CI: 73.1% to 85.5%) in the rabeprazole group, with a difference of 1.9% (95% CI: -6.7% to 10.5%, *P=*0.67). In the modified intention-to-treat (mITT) analysis, the eradication rates were 89.1% (95% CI: 83.1% to 93.2%) and 88.9% (95% CI: 82.7% to 93.1%), respectively (difference: 0.2%; 95% CI: -7.0% to 7.4%, *P=*0.95). In the per-protocol (PP) analysis, the eradication rates were 90.9% (95% CI: 85.2% to 94.6%) and 90.0% (95% CI: 83.9% to 94.0%), respectively (difference: 0.9%; 95% CI: -5.8% to 7.6%, *P=*0.80). Regarding safety, the incidence of adverse events was slightly higher in the anaprazole group compared to the rabeprazole group (22.50% vs. 15.00%), but the difference was not statistically significant (*P=*0.09). Medication adherence was similar between the two groups (*P=*0.60).

**Conclusion:**

The quadruple regimen consisting of anaprazole combined with amoxicillin, furazolidone, and bismuth is non-inferior to the rabeprazole-based quadruple regimen for the eradication of *H. pylori* infection. There were no significant differences between the two groups in terms of eradication rate, incidence of adverse reactions, or medication adherence. Anaprazole, as an acid-suppressive agent, achieves satisfactory efficacy in *H. pylori* eradication therapy and holds promise for clinical application.

## 1 Introduction

*Helicobacter pylori* (*H. pylori*) is a Gram-negative, microaerophilic bacterium that colonizes the gastric mucosa, affecting approximately half of the global population (Li et al., 2023). As a major causative agent of chronic gastritis, peptic ulcer disease, gastric mucosa-associated lymphoid tissue lymphoma, and gastric cancer (Crowe, 2019), *H. pylori* infection has become a significant public health concern. A nationwide epidemiological survey conducted by the National Early Gastrointestinal Cancer Prevention and Treatment Center reported an *H. pylori* infection rate of 40.66% in China, with rates of 43.45% in adults, 20.55% in children and adolescents, and 71.21% in households (Zhou et al., 2023). Accordingly, major international guidelines (Zhou et al., 2022; Malfertheiner et al., 2022) unequivocally recommend that all infected individuals without competing considerations should receive eradication therapy.

Currently, bismuth-containing quadruple therapy, consisting of two antibiotics combined with bismuth and a proton pump inhibitor, is the primary first-line empirical treatment regimen (Zhou et al., 2022). The selection of sensitive antibiotics is a critical determinant of eradication success. A meta-analysis published in 2024 in China reported resistance rates of 70.14% for metronidazole, 30.72% for clarithromycin, and 32.98% for levofloxacin. In marked contrast, resistance rates for amoxicillin, tetracycline, and furazolidone were only 2.41%, 2.53%, and 1.54%, respectively (Zeng et al., 2024). Susceptibility data from the Nanjing region further confirmed that resistance to furazolidone and amoxicillin was as low as 0.19% and 0.51%, respectively, while resistance to clarithromycin and metronidazole reached 50.01% and 84.45% (Wang et al., 2026a). Based on these resistance profiles, the combination of amoxicillin and furazolidone offers distinct advantages. Amoxicillin is a pH-dependent antibiotic, when intragastric pH rises from 5.5 to 7.5, its minimum inhibitory concentration against *H. pylori* decreases from 0.5 μg/mL to 0.06 μg/mL, significantly enhancing its bactericidal activity (Gao et al., 2020). Furazolidone, a nitrofuran antibiotic, exerts its antibacterial effect by interfering with bacterial oxidoreductase systems and is characterized by extremely low resistance rates and no cross-resistance with other antibiotics (Chen et al., 2022). Although furazolidone is primarily indicated for refractory *H. pylori* infection, *2022 chinese national clinical practice guideline on helicobacter pylori eradication treatment* (Zhou et al., 2022) explicitly states that in regions with high resistance to clarithromycin, levofloxacin, and metronidazole (including Jiangsu Province), or in patients with prior exposure to macrolides, quinolones, and nitroimidazoles, where eradication is anticipated to be challenging, a bismuth-containing quadruple regimen including furazolidone may be used. Among the recommended antibiotic combinations in this context is the use of amoxicillin combined with furazolidone.

In addition to selecting appropriate antibiotics, the degree of acid suppression provided by proton pump inhibitors is a key determinant of eradication success. PPIs enhance antibiotic activity by increasing intragastric pH, which converts *H. pylori* from a dormant to a replicative state (Labenz, 2001). Ke et al. (2021) first demonstrated that successful eradication with bismuth-containing quadruple therapy requires a median 24-hour intragastric pH of 5.7 or higher. However, the pharmacokinetics of conventional PPIs are substantially influenced by CYP2C19 genetic polymorphisms, and rapid metabolizers frequently experience treatment failure due to inadequate acid suppression. Rabeprazole, a classic second-generation PPI, is characterized by rapid onset and relatively limited CYP2C19 effects (Hu et al., 2005). Nonetheless, given the growing clinical demand for higher eradication rates, the pursuit of newer acid suppressants with more stable and consistent efficacy remains a priority for treatment optimization.

Anaprazole is a novel, domestically developed benzimidazole-class PPI that has been approved by the National Medical Products Administration for the treatment of duodenal ulcers. It has a unique pharmacokinetic profile, with clearance mediated by both non-enzymatic pathways (49%) and the cytochrome P450 enzyme system (51%), among which CYP3A4 is the major metabolic enzyme (contributing 48.3%), while CYP2C19-mediated metabolism accounts for only 3.5% (Ke et al., 2021; Liu et al., 2023). This profile minimizes the impact of CYP2C19 genetic polymorphisms on acid suppression, ensuring more consistent efficacy and a lower risk of drug-drug interactions. Phase II and III non-inferiority trials for duodenal ulcers have demonstrated that its acid-suppressive effect is comparable to that of rabeprazole (Zhu et al., 2022; Shu et al., 2021). Pharmacokinetic modeling studies have also shown that the risk of drug-drug interactions remains manageable when anaprazole is used in combination with amoxicillin and clarithromycin for *H. pylori* eradication (Liang et al., 2023).

In summary, in the context of rising antimicrobial resistance, a bismuth-containing quadruple regimen comb+ining low-resistance antibiotics (amoxicillin and furazolidone) with the novel PPI anaprazole may theoretically further improve *H. pylori* eradication rates by providing more stable and potent acid suppression. However, prospective studies evaluating the efficacy and safety of this regimen are currently lacking. To address this gap, we plan to conduct a prospective, multicenter, randomized, open-label, non-inferiority clinical trial in Jiangsu Province, using the classic PPI rabeprazole as the comparator, to assess the clinical value of anaprazole-based quadruple therapy for *H. pylori* eradication and to provide new evidence for optimizing eradication strategies in China. To avoid suboptimal eradication rates due to insufficient PPI dosing, we adopted a 20 mg twice-daily regimen, which is commonly used in clinical studies, to ensure all patients within the group receive stable and adequate acid suppression. This approach is expected to maximize antibiotic efficacy and minimize confounding variables. It should be noted that this study did not directly evaluate the mechanistic advantages of anaprazole in acid suppression. Its novelty lies in being the first randomized clinical trial of anaprazole-based quadruple therapy for *H. pylori* eradication, rather than in demonstrating a mechanistic advantage in acid inhibition.

## 2 Materials and methods

### 2.1 Study overview

This trial employed a prospective, multicenter, open-label, non-inferiority design, with methodological details consistent with our previously reported framework (Wang et al., 2026b). A total of 320 treatment-naïve patients diagnosed with *H. pylori* infection at the gastroenterology outpatient departments of four hospitals in Jiangsu Province (Nanjing First Hospital, Lianshui County People’s Hospital, The First People’s Hospital of Suqian and Changzhou Traditional Chinese Medicine Hospital) between August 2025 and February 2026 were enrolled. The study was approved by the Medical Ethics Committee of Nanjing First Hospital Affiliated to Nanjing Medical University (Approval No. KY20250722-11), registered with the Chinese Clinical Trial Registry (ChiCTR2600117235), and conducted in accordance with the Declaration of Helsinki. All interventions involved commercially available medications, and all patients were fully informed of the potential risks and provided written informed consent.

### 2.2 Study participants

#### 2.2.1 The inclusion criteria included:

1. Age between 18 and 65 years;
2. Confirmed current *H. pylori* infection, as evidenced by positive results on both the ^13^C-urea breath test and stool antigen test, with no prior history of eradication therapy;
3. Voluntary participation in the trial and provision of written informed consent.

#### 2.2.2 The exclusion criteria were:

1. Allergy to any study medication;
2. Recent gastroduodenal ulcer bleeding or evidence of bleeding within 4 weeks;
3. Prior history of *H. pylori* eradication therapy;
4. Use of antibiotics or bismuth agents within 4 weeks, or use of H_2_-receptor antagonists or PPIs within 2 weeks before treatment initiation;
5. Current use of corticosteroids, nonsteroidal anti-inflammatory drugs, or anticoagulants;
6. History of esophageal or gastric surgery;
7. Pregnancy or lactation;
8. Presence of other serious physical or mental disorders, or hepatic or renal insufficiency;
9. Gastric mucosa-associated lymphoid tissue lymphoma or other malignant diseases;
10. Glucose-6-phosphate dehydrogenase deficiency or bronchial asthma;
11. Concomitant use of specific medications: atazanavir or rilpivirine, tricyclic antidepressants, levodopa, acetaminophen, guaifenesin/dextromethorphan, monoamine oxidase inhibitors, tramadol, or similar agents.

#### 2.2.3 Sample size calculation

Based on previously published efficacy data for rabeprazole-based quadruple therapy, we assumed an eradication rate of 90% for both the rabeprazole and anaprazole quadruple groups. To demonstrate the non-inferiority of anaprazole-based quadruple therapy compared with rabeprazole-based quadruple therapy, a non-inferiority margin of -10% was set, with a one-sided significance level of 0.025. To achieve 80% power, a sample size of 142 participants per group was calculated. Accounting for potential loss to follow-up and refusal, a minimum of 160 participants per group was required. Accordingly, a total of 320 participants (160 per group) were enrolled in this study.

The selection of the -10% non-inferiority margin was based on the clinical context of PPI-based bismuth-containing quadruple therapy and the established efficacy of rabeprazole as the comparator. Rabeprazole-based bismuth-containing quadruple therapy is a first-line regimen recommended by Chinese guidelines, with an expected eradication rate of approximately 90% in this population. This study primarily investigated whether anaprazole, a novel PPI with distinct metabolic characteristics, could maintain this established level of efficacy when substituted for rabeprazole while all other medications remained identical.

A −10% margin was considered the maximum clinically acceptable loss of efficacy for this specific PPI-versus-PPI comparison. Because both arms received identical antibiotics and bismuth, the only variable was the PPI component. A loss exceeding 10% would indicate that anaprazole provides clinically inferior acid suppression for supporting antibiotic activity. Conversely, a loss within 10% would not outweigh the potential advantages of anaprazole, which remain relevant even within a quadruple regimen. Previous multicenter, randomized, double-blind, non-inferiority trials of bismuth-containing quadruple therapy for first-line *H. pylori* eradication have employed a similar margin, demonstrating that a loss of efficacy within 10% does not compromise the clinical utility of a regimen that maintains the same antibiotic backbone and bismuth component (Xie et al., 2023).

#### 2.2.4 Randomization procedure

##### 2.2.4.1 Randomization

Participants were randomized using a block randomization method with a block size of 4. A professional biostatistician generated the randomization sequence using SAS software (version 9.3). Eligible participants were assigned in a 1:1 ratio with equal probability to either the Anaprazole Group or the Rabeprazole Group.

##### 2.2.4.2 Group assignment

Eligible patients with *H. pylori* infection who provided informed consent by reading and checking the consent box completed a baseline information questionnaire on the Wenjuanxing platform. The questionnaire covered general information, personal history, lifestyle factors, and medical history. The platform backend assigned participants sequentially to the pre-generated randomization sequence based on the order of questionnaire submission and displayed the assigned group directly on the participant’s mobile device after submission. Investigators then administered the corresponding treatment regimen according to this group assignment.

### 2.3 Study procedures

#### 2.3.1 Intervention and grouping

Eligible participants were randomly assigned in a 1:1 ratio to one of two treatment groups: the Anaprazole Group or the Rabeprazole Group. Patients in the Anaprazole Group received anaprazole 20 mg and colloidal bismuth pectin 300 mg 30 minutes before breakfast and dinner, and amoxicillin 1.0 g and furazolidone 100 mg 30 minutes after breakfast and dinner. Patients in the Rabeprazole Group received rabeprazole 20 mg and colloidal bismuth pectin 300 mg 30 minutes before breakfast and dinner, and amoxicillin 1.0 g and furazolidone 100 mg 30 minutes after breakfast and dinner. Both regimens were administered for 14 days.

#### 2.3.2 Data collection and follow-up

Before treatment initiation, all participants completed an electronic questionnaire to provide personal information, including demographic and clinical characteristics, for baseline data collection. To minimize recall bias, antibiotic use over the previous two years was collected using a visual-assisted recall method. A color-illustrated booklet containing the names and images of commonly used antibiotics was developed. At enrollment, participants were shown the images and names of each antibiotic class and asked to identify those they had used.

During the 14-day eradication therapy, all participants received instructions on medication use and precautionary measures. Within three days after treatment completion, study personnel conducted follow-up via WeChat or telephone to document adverse events and medication adherence. At four weeks after treatment completion, participants were contacted and asked to return to the hospital for a ^13^C-urea breath test to evaluate *H. pylori* eradication status.

#### 2.3.3 Outcome measures

The primary efficacy outcome was the *H. pylori* eradication rate. Eradication success was defined as a negative urea breath test result at least 28 days after the last dose. The secondary efficacy outcomes included: (1) Medication adherence, quantified as the percentage of actual pills taken relative to the total prescribed over the 14-day course (14 days × daily dosing frequency), with ≥80% defined as good adherence and <80% as poor adherence. (2) Incidence of adverse events, summarized based on participant self-reports and follow-up data. Any abnormal health conditions occurring during the treatment period were recorded.

#### 2.3.4 Multicenter quality control

To ensure rigor and reproducibility, all participating centers strictly adhered to standardized operating procedures. Before study initiation, the project team developed a detailed standardized operation manual covering uniform inclusion and exclusion criteria, data collection methods, follow-up procedures, and intervention protocols. All research personnel received standardized training through online meetings before enrollment. All clinical centers used the same batches of medications from the same manufacturers, as follows: amoxicillin (Amoxicillin Capsules, Hunan Kelun Pharmaceutical Co., Ltd., National Drug Approval No. H43022210), furazolidone (Furazolidone Tablets, Tianjin Lisheng Pharmaceutical Co., Ltd., National Drug Approval No. H12020160), anaprazole (Anaprazole Sodium Enteric-coated Tablets, Beijing Jingfeng Pharmaceutical Group Co., Ltd., National Drug Approval No. H20230014), rabeprazole (Rabeprazole Sodium Enteric-coated Tablets, Zhuhai Rundu Pharmaceutical Co., Ltd., National Drug Approval No. H20234543), and bismuth (Colloidal Bismuth Pectin for Suspension, Hunan Warner Pharmaceutical Co., Ltd., National Drug Approval No. H20052104). To ensure consistency and accuracy in data collection, all centers used the same electronic questionnaire via the Wenjuanxing platform. The questionnaire included necessary range validations and required fields to minimize data entry errors and missing values. Standardized electronic forms were exported from the platform by researchers for subsequent unified data analysis.

### 2.4 Statistical analysis

The primary efficacy endpoint was assessed across three analysis populations. The intention-to-treat (ITT) population comprised all randomized individuals, with missing post-treatment urea breath test results imputed as eradication failures, irrespective of medication intake or follow-up completion. The modified intention-to-treat (mITT) population included all randomized participants who received at least one dose of the study medication and returned for a valid urea breath test at the designated follow-up time point, regardless of the level of regimen compliance. The per-protocol (PP) population was restricted to those who completed the follow-up assessment and maintained good treatment adherence, defined as consumption of 80% or more of the prescribed total doses.

Continuous data were summarized as means and standard deviations and compared using Student’s t-test for independent samples. Categorical variables were expressed as counts with percentages and analyzed using either the chi-square test or Fisher’s exact test, as appropriate. To establish non-inferiority of the anaprazole-containing regimen relative to the rabeprazole-based control, we employed a one-sided Z-test for the risk difference, along with corresponding 95% confidence intervals. Binary logistic regression was conducted to explore potential associations between baseline demographic and clinical variables, the occurrence of adverse events, and the likelihood of *H. pylori* eradication failure. For all logistic regression models, the dependent variable was coded as follows: eradication failure (positive ¹³C-urea breath test at the designated follow-up) = 1, and eradication success (negative ¹³C-urea breath test) = 0. Therefore, an odds ratio (OR) > 1 indicates a higher odds of eradication failure, whereas an OR < 1 indicates a lower odds of eradication failure.

In **Table 1**, all ORs, 95% confidence intervals, and P-values were derived from univariate binary logistic regression models using the Wald test. These P-values were not obtained from separate chi-square or Fisher’s exact tests. Variables with zero events in either the success or failure category could not be reliably estimated in the logistic regression model and were therefore not included in the model. For variables with sparse data (e.g., diabetes, with 3 successes and 2 failures), the asymptotic Wald test may be less reliable, and the corresponding OR and 95% confidence interval should be interpreted with caution. The wide confidence interval reflects the limited number of events rather than a true absence of association.

**Table 1:** Regression analysis of factors influencing *H. pylori* eradication rates.

Variables	Eradication success (n=256)	Eradication failure (n=27)	P-value	OR (95% CI)
Sex (Male/Female)				
Male	112(43.75%)	15(55.56%)	Reference	Reference
Female	144(56.25%)	12(44.44%)	0.24	0.622(0.280 ~ 1.382)
Age (years)	42.15 ± 12.51	42.85 ± 11.62	0.77	1.032(0.829 ~ 1.284)
BMI(kg/m2)	24.08 ± 3.56	24.13 ± 2.81	0.93	1.008(0.810 ~ 1.254)
Family size				
<3 persons	39(15.23%)	5 (18.52%)	Reference	Reference
≥3 persons	217(84.77%)	22 (81.48%)	0.66	0.791(0.283 ~ 2.213)
Long-term residence				
Urban	239(93.36%)	26 (96.30%)	0.56	1.849(0.237 ~ 14.46)
Rural	17 (6.64%)	1 (3.70%)	Reference	Reference
Drinking water type				
Tap water	155(60.55%)	12 (44.44%)	0.11	0.521(0.234 ~ 1.159)
Purified water	79(30.86%)	13 (48.15%)	0.07	2.080(0.934 ~ 4.628)
Mineral water	22(8.59%)	2 (7.41%)	0.83	0.851(0.189 ~ 3.834)
Well water	0(0.00%)	0 (0.00%)	–	–
Medical history				
Diabetes	3(1.17%)	2 (7.41%)	0.07	6.747(1.076 ~ 42.31)
Hypertension	35(13.67%)	4 (14.81%)	0.87	1.098(0.358 ~ 3.366)
Coronary heart disease	4(1.56%)	0 (0.00%)	–	–
Hyperlipidemia	17(6.64%)	1 (3.70%)	0.55	0.541(0.069 ~ 4.229)
Fatty liver disease	37(14.45%)	2 (7.41%)	0.31	0.474(0.107 ~ 2.081)
Smoking history	52(20.31%)	8 (29.63%)	0.26	1.652(0.685 ~ 3.984)
Alcohol use	95(37.11%)	12 (44.44%)	0.46	1.356(0.609 ~ 3.018)
Primary dining method				
Individual meals	35(13.67%)	5 (18.52%)	0.49	1.435(0.510 ~ 4.039)
Family meals	196(76.56%)	21 (77.78%)	0.89	1.071(0.413 ~ 2.777)
Dining out	25(9.77%)	1 (3.70%)	0.32	0.355(0.046 ~ 2.732)
Frequency of dining out with multiple people				
Rare (<1 time/month)	103(40.23%)	13 (48.15%)	0.43	1.379(0.623 ~ 3.057)
Seldom (<1 time/week)	66(25.78%)	9 (33.33%)	0.40	1.439(0.617 ~ 3.360)
Occasionally (1–3 times/week)	55(21.48%)	4 (14.81%)	0.42	0.636(0.211 ~ 1.917)
Often (>3 times/week)	32(12.50%)	1 (3.70%)	0.21	0.269(0.035 ~ 2.053)
Gastrointestinal symptoms				
No	116(45.31%)	12 (44.44%)	Reference	Reference
Yes	140(54.69%)	15 (55.56%)	0.94	1.036(0.466 ~ 2.300)
Family history of infection				
Not tested	137(53.52%)	16 (59.26%)	0.57	1.263(0.565 ~ 2.830)
No	43(16.80%)	3 (11.11%)	0.45	0.619(0.178 ~ 2.149)
Yes	76(29.69%)	8(29.63%)	1.00	0.997(0.418 ~ 2.377)
History of antibiotic use in past 2 years				
Amoxicillin/Penicillin	93(36.33%)	6(22.22%)	0.15	0.501(0.195 ~ 1.285)
Clarithromycin	4(1.56%)	2(7.41%)	0.10	5.040(0.879 ~ 28.88)
Metronidazole/Tinidazole	25(9.77%)	1(3.70%)	0.30	0.355(0.046 ~ 2.732)
Levofloxacin	17(6.64%)	1(3.70%)	0.55	0.541(0.069 ~ 4.229)
Furazolidone	0(0.00%)	0(0.00%)	–	–
Tetracycline	0(0.00%)	0(0.00%)	–	–
Cephalosporins	77(30.08%)	7(25.93%)	0.65	0.814(0.330 ~ 2.004)
Other/Unknown	11(4.30%)	4(14.81%)	0.02	3.874(1.142 ~ 13.15)
Adverse Events	37(14.45%)	5(18.52%)	0.57	1.345(0.480 ~ 3.774)

Non-inferiority testing was performed using SAS software (version 9.3, SAS Institute Inc.). All other statistical analyses were performed using IBM SPSS Statistics (version 27.0.1). A significance level of α= 0.05 was set, and a P-value <0.05 was considered statistically significant.

## 3 Results

### 3.1 Participant enrollment

Between August 2025 and February 2026, a total of 320 eligible participants who met the inclusion criteria were enrolled and included in the ITT analysis set. The study flow diagram is shown in **Figure 1**.

**Figure 1:**
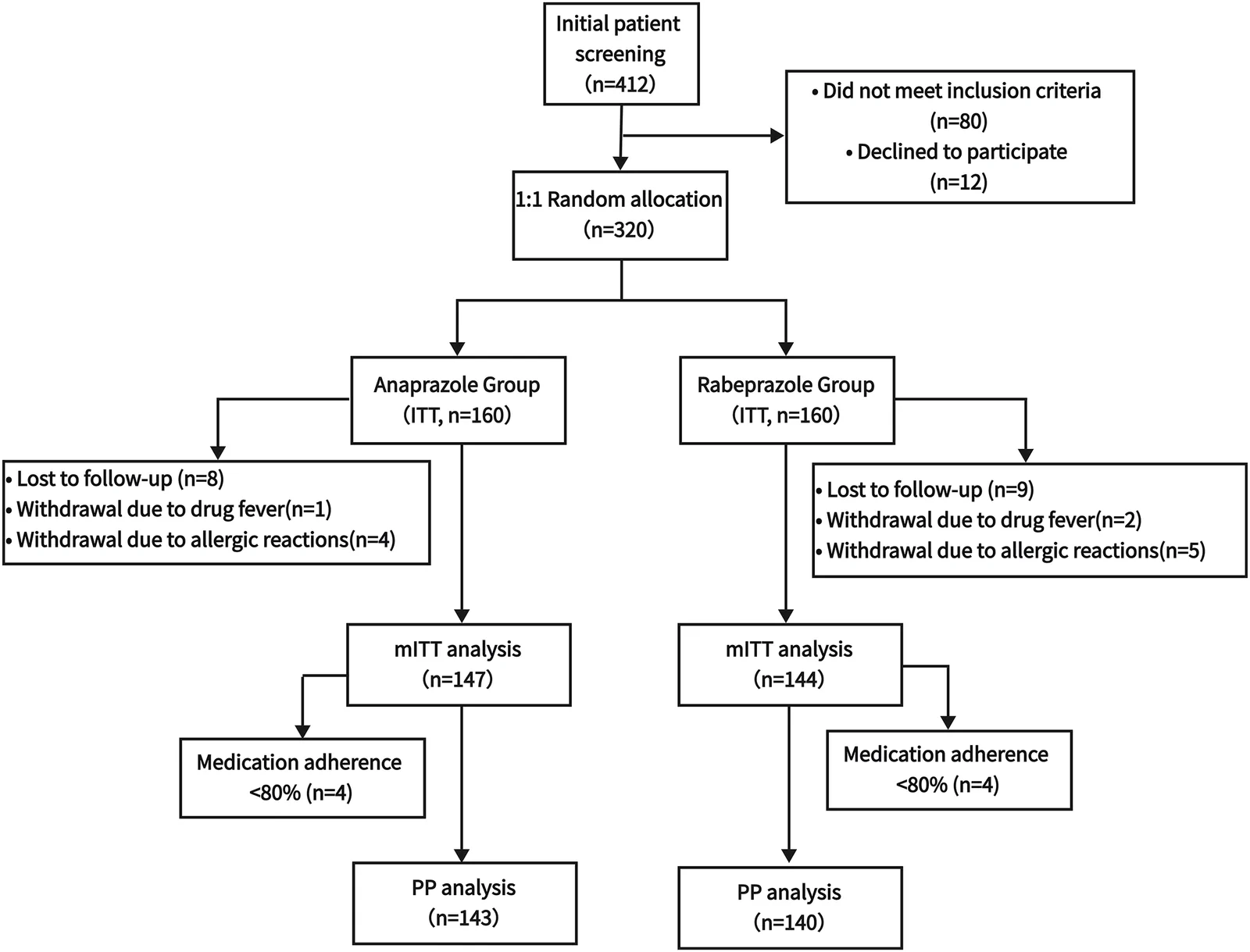
Study Flow Diagram.

### 3.2 Baseline characteristics

The baseline characteristics of all 320 participants are presented in **Table 2**. There were no significant differences between the two groups in age, sex, or body mass index (all P>0.05). The majority of participants had a household size of ≥3 (P = 0.42), long-term urban residence (P = 0.08), and tap water as the primary drinking water source (P = 0.37), with no differences between groups. No between-group differences were observed in the prevalence of comorbidities, including diabetes (P = 1.00), hypertension (P = 0.35), hyperlipidemia (P = 0.51), and fatty liver disease (P = 0.75). Smoking history (P = 0.35), alcohol consumption (P = 1.00), dining habits (P = 0.69), frequency of dining out (P = 0.92), and gastrointestinal symptoms (P = 0.73) also showed no significant differences. Family history of *H. pylori* infection (P = 0.70) and antibiotic use history, including amoxicillin/penicillin (P = 0.15), clarithromycin (P = 0.45), metronidazole/tinidazole (P = 0.84), levofloxacin (P = 0.66), cephalosporins (P = 0.14), and other/unknown antibiotics (P = 0.74), did not differ significantly between groups. No participants in either group had a history of furazolidone or tetracycline use within the preceding two years.

**Table 2:** Baseline demographic and clinical characteristics of the enrolled participants.

Variables	Anaprazole group (n=160)	Rabeprazole group (n=160)	P-value
Sex (Male/Female)	82/78	69/91	0.15
Age (years)	42.08 ± 11.79	40.27 ± 12.10	0.18
BMI(kg/m2)	23.62 ± 3.14	23.57 ± 3.19	0.89
Family size			0.42
<3 persons	26(16.3%)	20(12.5%)	
≥3 persons	134(83.8%)	140(87.5%)	
Long-term residence			0.08
Urban	153(95.6%)	144(90.0%)	
Rural	7(4.4%)	16(10.0%)	
Drinking water type			0.37
Tap water	88(55.0%)	97(60.6%)	
Purified water	55(34.4%)	51 (31.9%)	
Mineral water	16(10.0%)	12(7.5%)	
Well water	1(0.6%)	0(0.0%)	
Medical history			
Diabetes	4(2.5%)	3(1.9%)	1.00
Hypertension	27(16.9%)	21(13.1%)	0.35
Coronary heart disease	2(1.3%)	2(1.3%)	1.00
Hyperlipidemia	13(8.1%)	9 (5.6%)	0.51
Fatty liver disease	24(15.0%)	21( 13.1%)	0.75
Smoking history	38(23.8%)	30( 18.8%)	0.35
Alcohol use	65(40.6%)	65 (40.6%)	1.00
Primary dining method			0.69
Individual meals	23(14.4%)	22(13.8%)	
Family meals	121(75.6%)	126(78.8%)	
Dining out	16(10.0%)	12(7.5%)	
Frequency of dining out with multiple people			0.92
Rare (<1 time/month)	64(40.0%)	65(40.6%)	
Seldom (<1 time/week)	45(28.1%)	41 (25.6%)	
Occasionally (1–3 times/week)	31(19.4%)	34 (21.3%)	
Often (>3 times/week)	20(12.5%)	20 (12.5%)	
Gastrointestinal symptoms			0.73
No	75(46.9%)	79(49.4%)	
Yes	85(53.1%)	81(50.6%)	
Family history of infection			0.70
Not tested	85(53.1%)	91(56.9%)	
No	24(15.0%)	24(15.0%)	
Yes	51(31.9%)	45(28.1%)	
History of antibiotic use in past 2 years			
Amoxicillin/Penicillin	61(38.1%)	48(30.0%)	0.15
Clarithromycin	5(3.1%)	2(1.3%)	0.45
Metronidazole/Tinidazole	13(8.1%)	15(9.4%)	0.84
Levofloxacin	12(7.5%)	9(5.6%)	0.66
Furazolidone	0(0.0%)	0(0.0%)	–
Tetracycline	0(0.0%)	0(0.0%)	–
Cephalosporins	52(32.5%)	39(24.4%)	0.14
Other/Unknown	21(13.1%)	24(15.0%)	0.74

### 3.3 *H. pylori* eradication rates

The eradication rates for the Anaprazole Group and the Rabeprazole Group are presented in **Table 3**.

**Table 3:** Analysis of *H. pylori* eradication rates.

Outcome / Comparison	ITT (95%CI)	mITT (95%CI)	PP (95%CI)
Anaprazole Group	81.9%(131/160) (75.2%*～*87.1%)	89.1%(131/147) (83.1%*～*93.2%)	90.9%(130/143) (85.2%*～*94.6%)
Rabeprazole Group	80.0%(128/160) (73.1%*～*85.5%)	88.9%(128/144) (82.7%*～*93.1%)	90.0%(126/140) (83.9%*～*94.0%)
Risk Difference	1.9% (-6.7%*～*10.5%)	0.2% (-7.0%*～*7.4%)	0.9% (-5.8%*～*7.6%)
P-value for Difference	0.67	0.95	0.80
P-value for non-inferiority	<0.001	<0.001	<0.001

*Risk difference calculated as Anaprazole Group minus Rabeprazole Group. The prespecified non-inferiority margin was -10%. Non-inferiority is concluded if the lower limit of the 95% confidence interval for the risk difference is above -10%.

*H. pylori*, *Helicobacter pylori*; ITT, intention-to-treat; mITT, modified intention-to-treat; PP, per-protocol; CI, confidence interval.

In the ITT analysis, the eradication rate was 81.9% (95% CI: 75.2% to 87.1%) in the Anaprazole Group and 80.0% (95% CI: 73.1% to 85.5%) in the Rabeprazole Group, with no significant difference between the two groups (P = 0.67). In the non-inferiority analysis, the difference in eradication rates between the Anaprazole and Rabeprazole groups was 1.9% (95% CI: -6.7% to 10.5%). The non-inferiority P-value was <0.025, and the lower limit of the 95% CI (-6.7%) did not exceed the prespecified -10% non-inferiority margin.

In the mITT analysis, the eradication rate was 89.1% (95% CI: 83.1% to 93.2%) in the Anaprazole Group and 88.9% (95% CI: 82.7% to 93.1%) in the Rabeprazole Group, with no significant difference (P = 0.95). The non-inferiority analysis showed a difference of 0.2% (95% CI: -7.0% to 7.4%). The non-inferiority P-value was <0.025, and the lower limit of the 95% CI did not exceed the -10% margin.

In the PP analysis, the eradication rate was 90.9% (95% CI: 85.2% to 94.6%) in the Anaprazole Group and 90.0% (95% CI: 83.9% to 94.0%) in the Rabeprazole Group, with no significant difference (P = 0.80). The non-inferiority analysis revealed a difference of 0.9% (95% CI: -5.8% to 7.6%). The non-inferiority P-value was <0.025, and the lower limit of the 95% CI did not exceed the -10% margin.

### 3.4 Analysis of adverse effects and adherence

As shown in **Table 4**, in the ITT analysis, the overall incidence of adverse events was 22.50% (36/160) in the Anaprazole Group and 15.00% (24/160) in the Rabeprazole Group, with no significant difference between the two groups (P = 0.09). For specific adverse events, no statistically significant differences were observed between groups in the incidence of nausea/vomiting (3.75% vs. 2.50%, P = 0.75), diarrhea (2.50% vs. 1.25%, P = 0.68), abdominal bloating (2.50% vs. 1.25%, P = 0.68), rash (1.88% vs. 2.50%, P = 0.70), constipation (0.63% vs. 0, P = 1.00), abdominal pain (0.63% vs. 0, P = 1.00), Allergic reactions (2.50% vs. 3.13%, P = 0.74), Drug fever (0.63% vs. 1.25%, P = 1.00), or other adverse events(7.50% vs. 3.13%, P = 0.08). Medication adherence exceeded 87% in both groups, and no between-group difference was observed (P = 0.60).

**Table 4:** Analysis of adverse effects and medication adherence.

Adverse events	Anaprazole group (n=160)	Rabeprazole group (n=160)	P-value
Overall incidence	36(22.50%)	24(15.00%)	0.09
Diarrhea	4(2.50%)	2(1.25%)	0.68
Constipation	1(0.63%)	0(0.00%)	1.00
Nausea/Vomiting	6(3.75%)	4(2.50%)	0.75
Abdominal bloating	4(2.50%)	2(1.25%)	0.68
Abdominal pain	1(0.63%)	0(0.00%)	1.00
Rash	3(1.88%)	4(2.50%)	0.70
Allergic reactions	4(2.50%)	5(3.13%)	0.74
Drug fever	1(0.63%)	2(1.25%)	1.00
Other	12(7.50%)	5(3.13%)	0.08
Good adherence	143(89.38%)	140(87.50%)	0.60

### 3.5 Regression analysis of factors influencing *H. pylori* eradication rates

Univariate logistic regression analysis was performed to assess the influence of baseline characteristics, clinical features, and adverse events on the *H. pylori* eradication rate. In the PP population, the dependent variable was coded as eradication failure (positive ¹³C-urea breath test) = 1 and eradication success (negative ¹³C-urea breath test) = 0. Thus, OR > 1 indicate an increased odds of eradication failure, and OR < 1 indicate a decreased odds of eradication failure. Factors including sex, age, BMI, family size, long-term residence, drinking water type, medical history, smoking and alcohol use, primary dining method, frequency of dining out with multiple people, gastrointestinal symptoms, family history of infection, history of antibiotic use, and occurrence of adverse events were analyzed. As shown in **Table 1**, no significant associations with eradication outcome were found for sex (P = 0.24), age (P = 0.77), BMI (P = 0.93), family size (P = 0.66), long-term residence (P = 1.00), drinking water type (P = 0.19), diabetes history (P = 0.07), hypertension history (P = 0.87), coronary heart disease (P = 1.00), hyperlipidemia (P = 0.55), smoking (P = 0.26), alcohol use (P = 0.46), primary dining method (P = 0.50), frequency of dining out with multiple people (P = 0.39), gastrointestinal symptoms (P = 0.94), or family history of infection (P = 0.73). The occurrence of adverse events (P = 0.57) was not significantly associated with eradication success.

Regarding history of antibiotic use in the past 2 years, most specific antibiotic categories were not significantly associated with eradication failure, including amoxicillin/penicillin (P = 0.15), clarithromycin (P = 0.10), metronidazole/tinidazole (P = 0.30), levofloxacin (P = 0.55), and cephalosporins (P = 0.65). However, a history of other/unknown antibiotic use was significantly associated with an increased odds of eradication failure (OR 3.874, 95% CI 1.142–13.15, P = 0.02). Furazolidone and tetracycline use were not reported by any participant and could not be included in the model.

## 4 Discussion

Anaprazole is a novel, domestically developed benzimidazole-class PPI. Based on the PPI core structure, it incorporates a dihydrofuran ring and removes the methyl group at the R1 position of the pyridine ring, conferring several advantages. First, its metabolism involves both enzymatic and non-enzymatic pathways. Its clearance is mediated by non-enzymatic routes (49%) and the cytochrome P450 system (51%), with CYP3A4 being the major enzyme (48.3% contribution), while CYP2C19-mediated metabolism accounts for only 3.5% (Ke et al., 2021; Liu et al., 2023; Zhu et al., 2022). This multi-pathway metabolic profile theoretically ensures more stable acid suppression and lower interindividual variability. Second, anaprazole is excreted via both renal and biliary routes. Xie et al. (2024b) reported a total cumulative radioactive excretion rate of 93.20%, with urinary and fecal excretion rates of 53.34% and 39.86%, respectively. The total cumulative radioactive excretion within 96 hours after dosing was 91.6%, indicating high absorption and extensive metabolism, which may reduce renal burden and potentially enhance safety in patients with renal impairment. Third, anaprazole has a higher PKa value, enabling faster onset of action. Although anaprazole has demonstrated non-inferiority to rabeprazole in the treatment of duodenal ulcers (Zhu et al., 2022), and pharmacokinetic modeling has suggested a manageable risk of drug-drug interactions when combined with antibiotics (Liang et al., 2023), no prospective studies have yet evaluated its role in *H. pylori* eradication therapy. Therefore, this randomized controlled trial was designed to assess the efficacy and safety of anaprazole-based bismuth-containing quadruple therapy for *H. pylori* eradication.

Our results showed that the eradication rates of the anaprazole-based quadruple regimen in the ITT, mITT, and PP analysis sets were 81.9%, 89.1%, and 90.9%, respectively, closely matching those of the rabeprazole control group (80.0%, 88.9%, and 90.0%). The rate differences between the two groups ranged from 0.2% to 1.9%, and the lower limits of the 95% confidence intervals were far above the prespecified -10% non-inferiority margin (P<0.0001 for non-inferiority), confirming that the anaprazole-containing bismuth quadruple therapy is non-inferior to the rabeprazole-based regimen for *H. pylori* eradication. This result is consistent with the non-inferior performance of anaprazole in duodenal ulcer healing rates (Zhu et al., 2022), suggesting that its acid-suppressing potency may be sufficient to support the intragastric pH target required for H. pylori eradication (median pH ≥ 5.7) (Ke et al., 2021). However, because intragastric pH was not monitored in this study, this inference requires direct verification in further studies.

The appropriate selection of antibiotics is critical in *H. pylori* eradication therapy. Jiangsu Province and its surrounding areas are regions with high resistance to clarithromycin and levofloxacin, posing a substantial clinical challenge. Surveillance data from Nanjing between 2018 and 2024 showed primary resistance rates of 50.01% for clarithromycin and 38.59% for levofloxacin (Wang et al., 2026a). A nationwide meta-analysis further reported overall primary resistance rates of 30.72% and 32.98% for clarithromycin and levofloxacin, respectively (Zeng et al., 2024). *2022 chinese national clinical practice guideline on helicobacter pylori eradication treatment* (Zhou et al., 2022) explicitly states that in regions with high resistance to clarithromycin and levofloxacin, where eradication is anticipated to be challenging, a bismuth-containing quadruple regimen including amoxicillin and furazolidone may be used. Accordingly, our study selected the amoxicillin-furazolidone antibiotic combination based on regional resistance data and authoritative guideline recommendations.

Univariate logistic regression analysis in this study showed that various demographic characteristics, lifestyle factors, and clinical variables, including a history of amoxicillin use, were not significantly associated with eradication outcome. Xie et al. (2024a) reported that prior amoxicillin use significantly increases both the resistance rate and the minimum inhibitory concentration of *H. pylori* to amoxicillin, providing a mechanistic explanation for the association between prior antibiotic exposure and treatment failure. In addition, a study in refractory infection populations (Lin et al., 2025) confirmed that although the baseline resistance rate to amoxicillin is low, resistance increases significantly with each additional treatment course (OR = 1.60 per year), indicating that repeated exposure can induce amoxicillin resistance. A large-scale meta-analysis conducted in mainland China (Zeng et al., 2024) further demonstrated that although the overall primary resistance rate to amoxicillin is very low (2.41%), it rises substantially among individuals with prior antibiotic use. Our study did not identify amoxicillin use history as a risk factor for *H. pylori* eradication failure, which may be attributable to the limited sample size of the eradication failure group. Future studies should integrate prospective susceptibility testing, resistance gene detection, and larger sample sizes to elucidate the underlying mechanisms and to guide individualized treatment.

In this study, factors including age, sex, BMI, history of comorbidities, smoking and alcohol use, and dietary habits showed no significant association with eradication rate. This finding is consistent with some previous reports (Song et al., 2020), although evidence remains conflicting. Regarding the effect of smoking on *H. pylori* eradication, the literature is inconsistent. Although several studies and meta-analyses have identified smoking as an independent risk factor for eradication failure (Wu et al., 2024), Song et al. (2021) found that smokers had a significantly higher risk of failure (OR = 1.71, 95% CI: 1.17 to 2.50, P = 0.01). However, other studies have reported no significant association (Takara et al., 2019). In our study, smoking rates in the two groups were 23.8% and 18.8%, respectively, with no significant difference in distribution between the eradication success and failure subgroups. This may be due to the limited statistical power of the subgroup analysis given the sample size (n=320), or to unexamined heterogeneity in smoking intensity and duration. Future studies using standardized smoking indices, such as pack-years, may better quantify the impact of smoking. Similarly, the effect of alcohol consumption on eradication remains controversial. Some studies suggest that moderate alcohol intake may be beneficial for *H. pylori* clearance (Liu et al., 2016), though we did not observe a significant association in this study.

Regarding safety, the overall incidence of adverse events was 22.50% in the Anaprazole Group and 15.00% in the Rabeprazole Group, with no statistically significant difference between the two groups (P = 0.09). Common adverse events included nausea/vomiting, abdominal bloating, diarrhea, and rash, most of which were mild to moderate in severity and well tolerated, with no serious adverse events reported. Although the Anaprazole Group showed numerically higher incidences of nausea/vomiting (3.75% vs. 2.50%), none of these differences reached statistical significance. The overall safety profile was consistent with previous clinical data on anaprazole (Zhu et al., 2022). In terms of medication adherence, approximately 87% of patients in both groups took≥80% of the prescribed doses. This high adherence can be attributed to the simple 14-day, twice-daily regimen and stringent medication guidance. These findings indicate that, under standardized follow-up management, the anaprazole-containing bismuth quadruple therapy is well tolerated and associated with good adherence.

This study has several limitations. First, antimicrobial susceptibility testing was not performed. As we did not conduct *H. pylori* culture and susceptibility testing for enrolled patients, we could not distinguish the effects of primary versus secondary resistance on eradication outcomes. Although resistance rates to amoxicillin and furazolidone are very low in China, individual-level resistance may still exist. Future studies could incorporate susceptibility-guided individualized therapy to further optimize treatment outcomes. Second, the sample size was calculated primarily for non-inferiority testing and may have insufficient statistical power for risk factor analysis, particularly when the prevalence of certain factors (e.g., diabetes, smoking) is low, potentially leading to false-negative results. Furthermore, multivariable analysis was not performed to control for confounding factors. Future large-sample studies could adopt multivariable models to further evaluate the independent effects of each factor. Third, although this study adopted a multicenter design, all were located in Jiangsu Province, which may limit the generalizability of the findings. Given the vast geographical extent of China and regional variations in *H. pylori* resistance patterns, antibiotic selection strategies should be tailored to local conditions. Future studies conducted in broader geographical regions are needed to validate the generalizability of the anaprazole-based regimen. Fourth, the open-label design of this study was primarily chosen for operational feasibility. Although the primary outcome was assessed using an objective test and is unlikely to be influenced by this design, we acknowledge that the secondary outcomes relied on patient self-reporting, which may be subject to information bias. Specifically, patients who were aware that they were receiving the novel treatment may have been more inclined to report minor discomfort. Nevertheless, the differences in adverse events between the two groups did not reach statistical significance, and all reported events were mild to moderate in severity and well tolerated. Regarding adherence, over 87% of patients in both groups maintained good medication adherence, with no statistically significant difference between the groups (P = 0.60), suggesting that adherence-related bias likely had a limited impact on between-group comparisons. Future studies with a double-blind design and larger sample sizes would be valuable to further corroborate our findings. Fifth, a visual assisted recall method was used to collect data on participants’ antibiotic use over the previous two years. While this approach may reduce recall bias compared to unaided verbal questioning, it cannot fully ensure accurate recollection of the exact timing, dosage, or duration of antibiotic use. Because the study did not have access to Electronic Medical Records or a Drug Utilization Review system for cross verification, the antibiotic use data may still be subject to recall bias, which could have influenced the analysis of related variables.

## 5 Conclusion

The quadruple regimen consisting of anaprazole combined with amoxicillin, furazolidone, and bismuth is non-inferior to the rabeprazole-based quadruple regimen for the eradication of *H. pylori* infection. There were no significant differences between the two groups in terms of eradication rate, incidence of adverse reactions, or medication adherence. Anaprazole, as an acid-suppressive agent, achieves satisfactory efficacy in *H. pylori* eradication therapy and holds promise for clinical application.

## Data Availability

The raw data supporting the conclusions of this article will be made available by the authors, without undue reservation.
